# Polyphenols act synergistically with doxorubicin and etoposide in leukaemia cell lines

**DOI:** 10.1038/cddiscovery.2015.43

**Published:** 2015-11-23

**Authors:** AA Mahbub, CL Le Maitre, SL Haywood-Small, NA Cross, N Jordan-Mahy

**Affiliations:** 1 Department of Biosciences and Chemistry, Biomolecular Sciences Research Centre, Sheffield Hallam University, Floor 7, The Owen Building, Howard Street, Sheffield, South Yorkshire S1 1WB, UK

## Abstract

The study aimed to assess the effects of polyphenols when used in combination with doxorubicin and etoposide, and to determine whether polyphenols sensitised leukaemia cells, causing inhibition of cell proliferation, cell cycle arrest and induction of apoptosis. This study is based on findings in solid cancer tumours, which have shown that polyphenols can sensitize cells to chemotherapy, and induce apoptosis and/or cell-cycle arrest. This could enable a reduction of chemotherapy dose and off-target effects, whilst maintaining treatment efficacy. Quercetin, apigenin, emodin, rhein and *cis*-stilbene were investigated alone and in combination with etoposide and doxorubicin in two lymphoid and two myeloid leukaemia cells lines. Measurements were made of ATP levels (using CellTiter-Glo assay) as an indication of total cell number, cell cycle progression (using propidium iodide staining and flow cytometry) and apoptosis (NucView caspase 3 assay and Hoechst 33342/propidium iodide staining). Effects of combination treatments on caspases 3, 8 and 9 activity were determined using Glo luminescent assays, glutathione levels were measured using the GSH-Glo Glutathione Assay and DNA damage determined by anti-*γ*H2AX staining. Doxorubicin and etoposide in combination with polyphenols synergistically reduced ATP levels, induced apoptosis and increased S and/or G_2_/M phase cell cycle arrest in lymphoid leukaemia cell lines. However, in the myeloid cell lines the effects of the combination treatments varied; doxorubicin had a synergistic or additive effect when combined with quercetin, apigenin, emodin, and cis-stilbene, but had an antagonistic effect when combined with rhein. Combination treatment caused a synergistic downregulation of glutathione levels and increased DNA damage, driving apoptosis via caspase 8 and 9 activation. However, in myeloid cells where antagonistic effects were observed, this was associated with increased glutathione levels and a reduction in DNA damage and apoptosis. This study has demonstrated that doxorubicin and etoposide activity were enhanced by polyphenols in lymphoid leukaemia cells, however, differential responses were seen in myeloid cells with antagonistic responses seen in some combination therapies.

## Introduction

Despite considerable improvements in tolerance and efficacy of chemotherapeutic agents, the mortality of leukaemia is still high.^1^ Topoisomerase II inhibitors such as doxorubicin and etoposide are the most common chemotherapeutic agents used for leukaemia treatment.^1,2^ Unfortunately, these agents are commonly associated with severe side effects; and drug resistance is common.^1–6^ As such combination treatments are under investigation as they could enhance the efficacy of standard chemotherapy agents, and decrease development of drug resistance, toxicity and side effects.^1–3,5,7^

One strategy could be combination of chemotherapy agents with bioactive compounds such as polyphenols. We have previously shown that a number of polyphenols reduced cell ATP levels, caused cell cycle arrest and induce apoptosis, particularly in lymphoid leukaemia cells lines, whilst having limited effects on normal haematopoietic cells.^8^ Within our previous study, eight polyphenols selected as representatives of the major polyphenol classes were investigated, and the most potent polyphenols identified were quercetin, apigenin, emodin, rhein and *cis*-stilbene.^8^

In a number of solid tumours, polyphenols have been shown to work synergistically with cisplatin and doxorubicin and induce apoptosis.^3,9,10^ Thus, it may be possible for the polyphenols to enhance action of the standard chemotherapy treatments, however, no studies have investigated the combination effects of polyphenols with topoisomerase II inhibitors in leukaemia cells. This study investigated whether the polyphenols identified previously^8^ have a synergistic effect when combined with topoisomerase II inhibitor agents (doxorubicin and etoposide) on cell ATP levels, apoptosis and cell cycle progression within the lymphoid and myeloid leukaemia cell lines. The mechanism of action of these combination treatments was also investigated.

## Results

### Effects of topoisomerase inhibitors alone on ATP levels and caspase 3 activity in leukaemia cell lines

**Figure 1:**
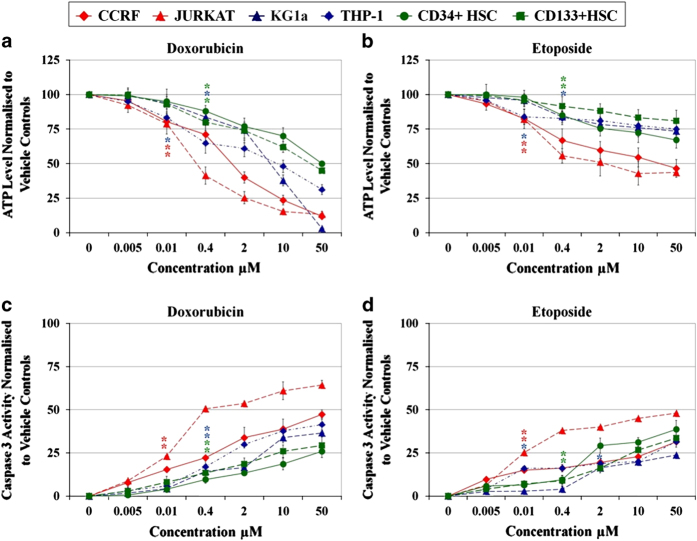
Effect of doxorubicin and etoposide on (**a** and **b**) ATP levels and (**c** and **d**) caspase 3 activity in two lymphoid leukaemia (CCRF-CEM and Jurkat), two myeloid leukaemia (THP-1 and KG-1a) cell lines and two non-tumour control cells (CD133^+^ HSC and CD34^+^ HSC). The lowest-significant doses (LSD) which significantly reduced ATP levels and induced apoptosis was determined for each etoposide inhibitor in each cell lines. The '*' indicated LSD in each cell line.

Doxorubicin and etoposide induced a dose-dependant decrease in ATP levels and increase in caspase 3 activity in all cell lines although effects on non-tumour control cells were reduced compared with leukaemia cells (Figure 1). The lowest-significant dose (LSD) for doxorubicin and etoposide alone (*μ*M), which reduced ATP levels compared with the vehicle control at 24 h, was dependant on all cell types. The LSD for ATP activity for both doxorubicin and etoposide were: 0.01 *μ*M in Jurkat; 0.01 *μ*M in CCRF-CEM; 0.01 *μ*M in THP-1; 0.4 *μ*M in KG-1a; 0.4 *μ*M in CD34^+^ HSCs and 0.4 *μ*M in CD133^+^ HSCs (Figures 1a and b). Similar LSDs were observed for induction of caspase 3 activity although this varied slightly dependant on chemotherapy agent and cell line investigated. For doxorubicin, the LSD for caspase 3 activity were: 0.01 *μ*M in Jurkat; 0.01 *μ*M in CCRF-CEM; 0.4 *μ*M in THP-1; 0.4 *μ*M in KG-1a; 0.4 *μ*M in CD34^+^ HSCs and 0.4 *μ*M in CD133^+^ HSCs (Figure 1c). For etoposide, the LSD for caspase 3 activity were: 0.01 *μ*M in Jurkat; 0.01 *μ*M in CCRF-CEM; 0.01 *μ*M in THP-1; 0.4 *μ*M in KG-1a; 0.4 *μ*M in CD34^+^ HSCs and 0.4 *μ*M in CD133^+^ HSCs (Figure 1d).

### Effect of Topoisomerase inhibitors in combination with polyphenols on ATP levels in leukaemia cell lines and non-tumour cells

**Figure 2:**
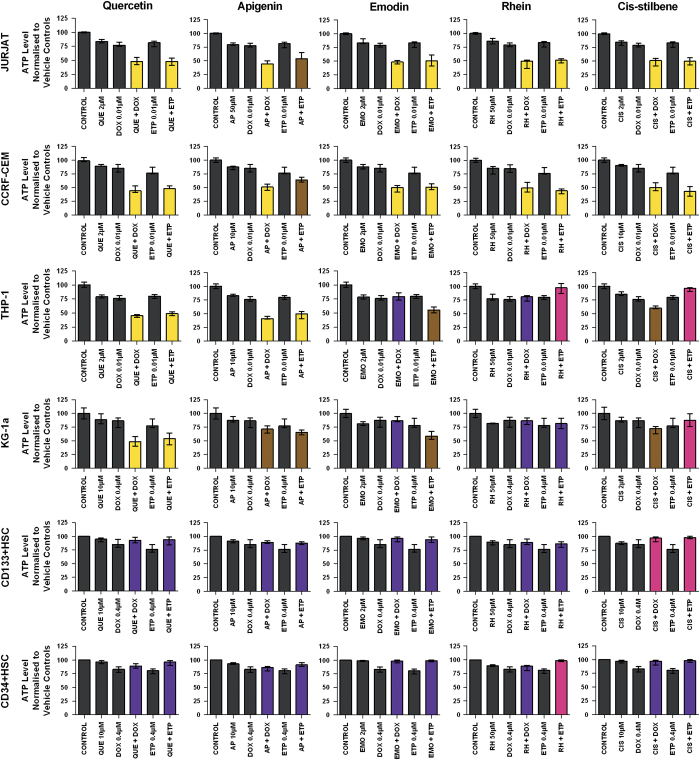
The effect of doxorubicin (DOX) and etoposide (ETP) when used in combination with quercetin (QUE), apigenin (AP), emodin (EMO), rhein (RH) or *cis*-stilbene (CIS) on ATP levels in two lymphoid leukaemia cell lines (Jurkat and CCRF-CEM), two myeloid leukaemia cell lines (THP-1 and KG-1a) and two non-tumour control cells (CD133+ HSC and CD34+ HSC). This was evaluated by CellTiter-Glo assay. Cells were treated with doxorubicin or etoposide and polyphenols alone and in combination for 24 h using their lowest-significant doses (LSD); together with a vehicle control. All data were normalised to the vehicle control, which was assigned 100% cell viability. The data were expressed as medians and ranges (*n*=4). The black bars show the vehicle controls and treatments alone; the coloured bars indicate significant additive effects in brown, synergistic effects in yellow, competitive antagonistic effects in purple and antagonistic effects in pink. Statistical significance was set at *P*⩽0.05 compared with vehicle control, drugs alone and expected values of individual drugs when combined.

Doxorubicin and etoposide when used in combination with quercetin caused a synergistic reduction of ATP levels (*P*<0.05) in both lymphoid and myeloid cell lines, however, minimal changes were found in non-tumour control cells (Figure 2). Doxorubicin combined with apigenin induced a synergistic reduction in ATP levels in three out of four leukaemia cell lines (CCRF-CEM, Jurkat and THP-1) and had an additive effect in KG-1a cells (*P*<0.05) (Figure 2). Etoposide when combined with apigenin induced a synergistic decrease in ATP levels in THP-1 myeloid cell line only, but had additive effects in all other leukaemia cell lines. Limited effects were seen in non-tumour control cells (*P*<0.05) (Figure 2).

A synergistic reduction in ATP levels in the lymphoid leukaemia cell lines was also seen when doxorubicin or etoposide was combined with emodin, rhein or *cis*-stilbene (*P*<0.05) (Figure 2). However, in the myeloid cell lines, when doxorubicin or etoposide was combined with emodin, rhein or *cis*-stilbene, no synergistic effects were seen and responses varied between additive, competitively antagonistic or antagonistic (Figure 2). In the non-tumour control cells both rhein and *cis*-stilbene antagonised the effects of both doxorubicin and etoposide (Figure 2).

### Combination therapy effects on caspase 3 activity

**Figure 3:**
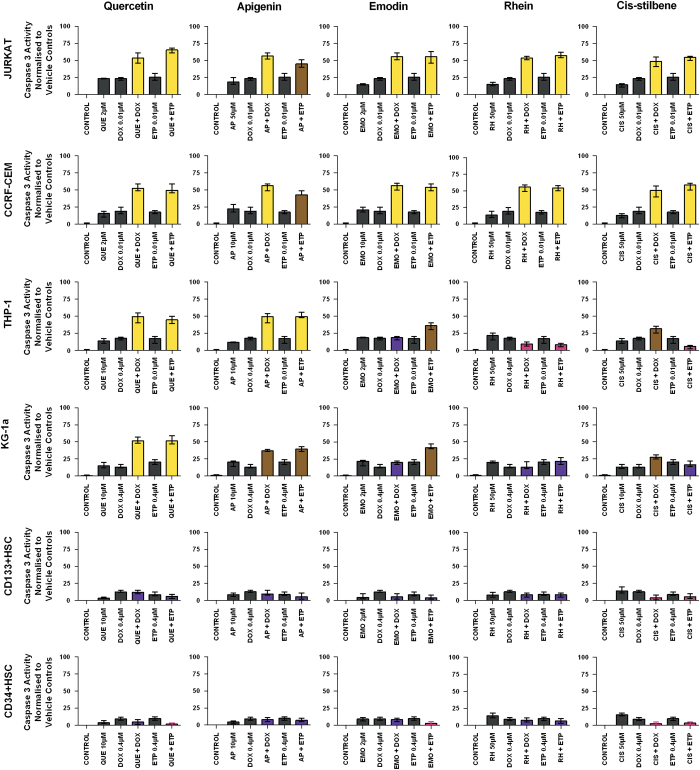
The effect of doxorubicin (DOX) and etoposide (ETP) when used in combination with quercetin (QUE), apigenin (AP), emodin (EMO), rhein (RH) or *cis*-stilbene (CIS) on caspase 3 activity of two lymphoid leukaemia cell lines (Jurkat and CCRF-CEM), two myeloid leukaemia cell lines (THP-1 and KG-1a) and two non-tumour control cells (CD133+ HSC and CD34+ HSC). This was evaluated by NucView caspase 3 activity assay. Cells were treated with doxorubicin or etoposide and polyphenols alone and in combination for 24 h using their lowest-significant doses (LSD), together with a vehicle control. All data were normalised to the vehicle control, which was assigned a 0% apoptotic level. The data were expressed as medians with ranges (*n*=4). The black bars show the vehicle controls and treatments alone; the coloured bars indicate significant additive effects in brown, synergistic effects in yellow, competitive antagonistic effects in purple and antagonistic effects in pink. Statistical significance was set at *P*⩽0.05 compared with vehicle control, drugs alone and expected values of individual drugs effects.

Doxorubicin and etoposide when used in combination with quercetin produced a synergistic increase in caspase 3 activity in all leukaemia cell lines (*P*<0.05) (Figure 3). Similarly, doxorubicin when used in combination with apigenin synergistically increased caspase 3 activity in three out of four of the leukaemia cell lines (CCRF-CEM, Jurkat and THP-1) (*P*<0.05), however, this combination only had an additive effect in KG-1a cells (*P*<0.05). A synergistic effect was observed when etoposide and apigenin were combined in the treatment of THP-1 cells and an additive effect was observed in the three remaining leukaemia cells lines (CCRF-CEM, Jurkat and KG-1a) (*P*<0.05) (Figure 3). Both doxorubicin and etoposide when used in combination with emodin, rhein or *cis*-stilbene synergistically increased caspase 3 activity in the lymphoid cell lines only (*P*<0.05) (Figure 3). In the myeloid cell lines, doxorubicin and etoposide when combined with emodin and *cis*-stilbene differently modulated caspase 3 activity, having either an additive, competitive-antagonistic or antagonistic effect (*P*<0.05) (Figure 3). In contrast, both doxorubicin and etoposide when combined with rhein produced either a competitive-antagonistic or antagonistic effect on apoptosis (*P*<0.05) (Figure 3). In the non-tumour control cells (CD34+ HSC and CD133+ HSC), both doxorubicin and etoposide when combined with each of the polyphenols had a minimal effect on caspase 3 activity (Figure 3). These results were further confirmed with morphological assessment of apoptosis, and were found to follow the same trends shown by the caspase 3 data (data not shown).

### Combination effect on caspases 8 and 9 activity

**Figure 4:**
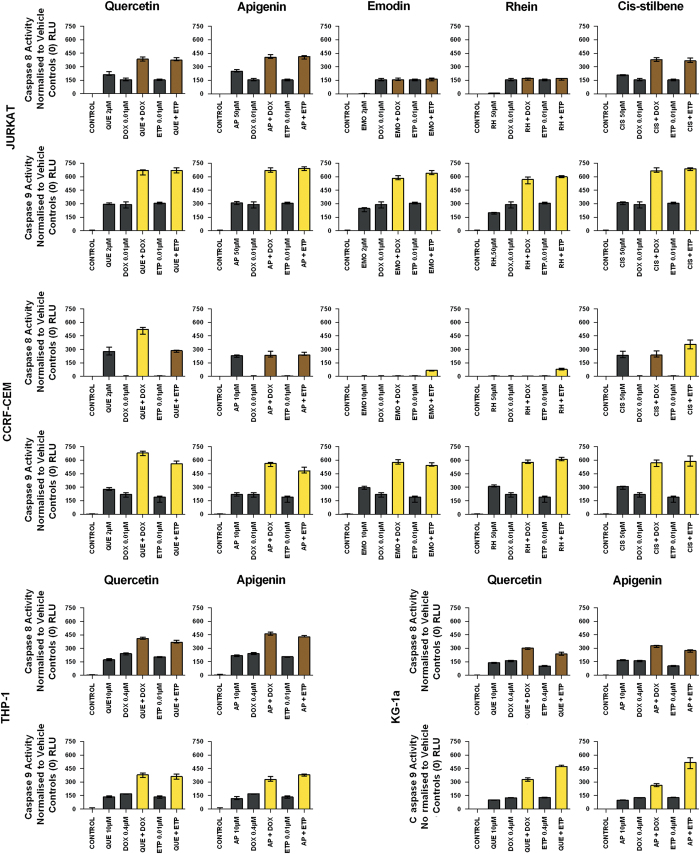
The effect of doxorubicin (DOX) and etoposide (ETP) on caspases 8 and 9 activity when used in combination with quercetin (QUE), apigenin (AP), emodin (EMO), rhein (RH) or *cis*-stilbene (CIS) in lymphoid leukaemia cell lines (Jurkat and CCRF-CEM); and when used in combination with QUE or AP in myeloid leukaemia cell lines (THP-1 and KG-1a). This was evaluated by Caspases-Glo Luminescent 8 and 9 Assays. Cells were treated with DOX or ETP and polyphenols alone and in combination for 24 h using their lowest-significant doses (LSD). Data were normalised to the vehicle control, which was assigned a 0 RLU. The data were expressed as medians with ranges (*n*=4). The black bars show the vehicle controls and treatments alone; the coloured bars indicate significant additive effects in brown, synergistic effects in yellow, competitive antagonistic effects in purple and antagonistic effects in pink. Statistical significance was set at *P*⩽0.05 compared with vehicle control, drugs alone and expected values of individual drugs effects.

Doxorubicin and etoposide when used in combination with each polyphenol caused a synergistic increase in caspase 9 activity (*P*<0.05) in all leukaemia cell lines (Figure 4). In contrast a synergistic effect on caspase 8 was only seen in CCRF-CEM cells when doxorubicin was combined with quercetin (*P*<0.05) or when etoposide was combined with emodin, rhein or *cis*-stilbene (*P*<0.05) (Figure 4). In all other combination treatments, in all other cell lines there was an additive increase in caspase 8 activity only (Figure 4); the only exception was in the CCRF-CEM cells when treated with doxorubicin and emodin or rhein which showed no caspase 8 activity.

### Combination effects on cell cycle progression

Within lymphoid leukaemia cell lines when doxorubicin or etoposide were combined with each of the polyphenol, interactive effects were observed with cells accumulating in the S and/or G_2_/M phase of the cell cycle (*P*<0.05) (Table 1). In myeloid cell lines, an interactive effect was observed when both doxorubicin and etoposide were combined with quercetin or apigenin in the THP-1 cells (*P*<0.05), and in KG1a cells, when etoposide was combined with apigenin: inducing an S and G_2_/M cell cycle arrest (*P*<0.05) (Table 1). In the THP-1 cells, when doxorubicin was in combination with *cis*-stilbene an interactive effect was seen with S phase arrest (*P*<0.05) (Table 1). In contrast, in the myeloid cell lines an antagonistic effect with no cell cycle arrest was seen when doxorubicin was combined with emodin or rhein, and when etoposide was combined with rhein and cis-stilbene (*P*<0.05) (Table 1). All other combination treatments in the myeloid cell lines showed no interactions between the polyphenols and topoisomerase inhibitors although cells accumulated in S and G_2_ phase of the cell cycle (Table 1).

### Glutathione levels and effects of combination treatments

**Figure 5:**
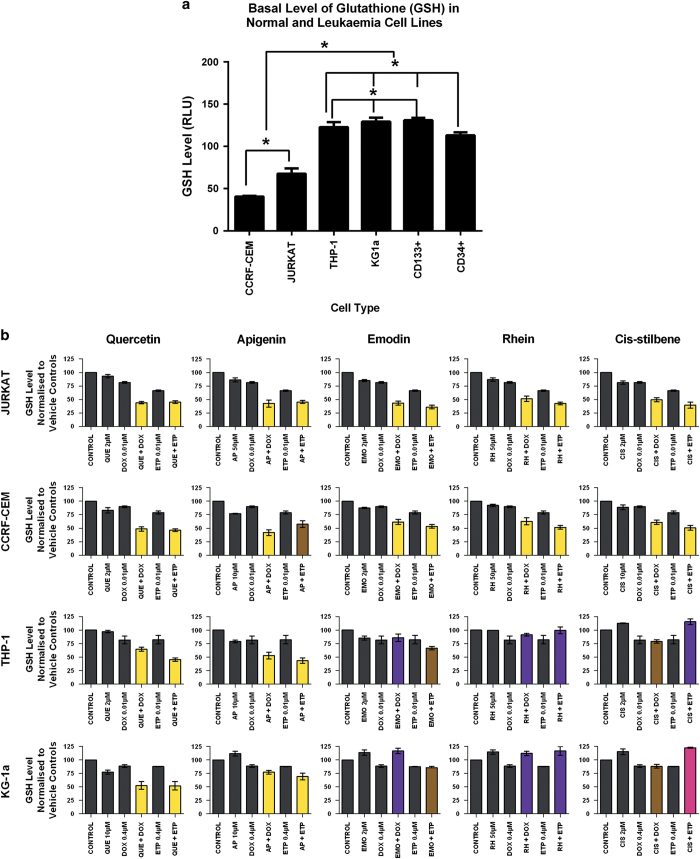
(**a**) Basal glutathione (GSH) levels in two lymphoid leukaemia (CCRF-CEM and JURKAT), two myeloid leukaemia (THP-1 and KG-1a) cell lines; and two non-tumour control (CD133^+^HSC and CD34^+^ HSC) cell lines. Untreated cells were evaluated by the GSH-Glo Glutathione Assay. The data are expressed as median with range in triplicate. Statistical significance was set at *P*⩽0.05. (**b**) The effect of doxorubicin (DOX) and etoposide (ETP) when used in combination with quercetin (QUE), apigenin (AP), emodin (EMO), rhein, (RH) or *cis*-stilbene (CIS) was determined on GSH levels in two lymphoid leukaemia cell lines (Jurkat and CCRF-CEM) and two myeloid leukaemia cell lines (THP-1 and KG-1a). GSH levels were evaluated by the GSH-Glo Glutathione Assay. Cells were treated with DOX or ETP and polyphenols alone and in combination for 24 h using their lowest-significant doses (LSD). Data were normalised to the vehicle control, which was assigned 100% of GSH level. The data were expressed as medians and ranges (*n*=4). The black bars show the vehicle controls and treatments alone; the coloured bars indicate a significant additive effect in brown, a synergistic effect in yellow, a competitive antagonistic effect in purple and an antagonistic effect in pink. Statistical significance was set at *P*⩽0.05 compared with vehicle control, drugs alone and expected values of individual drugs effects.

Basal glutathione levels varied between cells and these followed the following pattern: CCRF-CEM<Jurkat<CD34^+^ HSC<THP-1<KG-1a<CD133^+^ HSC (Figure 5a). Lymphoid leukaemia cell lines showed significantly lower glutathione levels compared with myeloid leukaemia cell lines and non-tumour control cells (*P*⩽0.05) (Figure 5a).

Following stimulation with doxorubicin and etoposide in combination with quercetin or apigenin there was a synergistic or additive decrease in glutathione levels in all lymphoid and myeloid cell lines (*P*<0.05) (Figure 5b). Similarly when doxorubicin and etoposide were used in combination with emodin, rhein and *cis*-stilbene this too caused a synergistic reduction in glutathione levels, but only in lymphoid leukaemia cell lines (*P*<0.05) (Figure 5b). In contrast in myeloid cell lines, when doxorubicin was combined with emodin and rhein, and etoposide was combined with rhein and *cis*-stilbene there was a competitive antagonistic or antagonist effect, and glutathione levels were increased (*P*<0.05) (Figure 5b). However, when etoposide was combined with emodin or *cis*-stilbene in myeloid cell lines there was an additive decrease in glutathione levels (*P*<0.05) (Figure 5b). These results were further confirmed using CMFDA staining, and were found to follow the same trends shown by the GSH-Glo Glutathione Assay (Promega, Southampton, UK; data not shown).

### Effects of combination of polyphenols and topoisomerase inhibitors on *γ*H2AX foci formation

**Figure 6:**
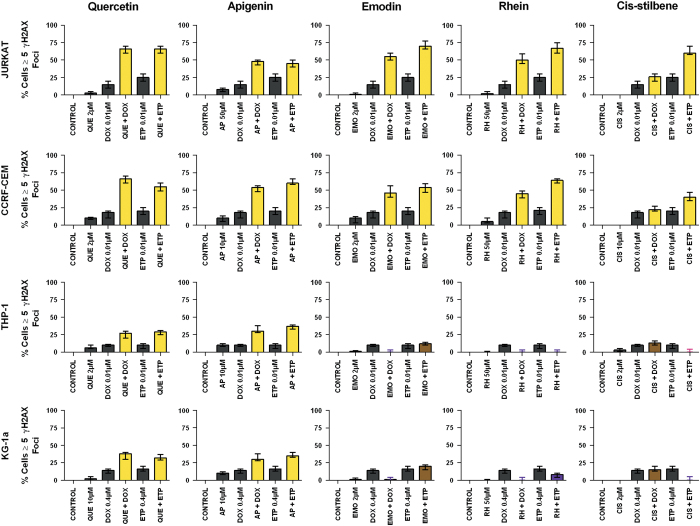
The effect of doxorubicin (DOX) and etoposide (ETP) when used in combination with quercetin (QUE), apigenin (AP), emodin (EMO), rhein, (RH) or *cis*-stilbene (CIS) on *γ*-H2AX foci formation (DNA damage marker) of two lymphoid leukaemia cell lines (Jurkat and CCRF-CEM) and two myeloid leukaemia cell lines (THP-1 and KG-1a). This was evaluated by the immunofluorescent staining using Alexa Fluor 647 Mouse anti-H2AX (pS139). Cells were treated with DOX or ETP and polyphenols alone and in combination for 24 h using their lowest-significant doses (LSD). The data were expressed as medians and ranges (*n*=4). The black bars show the vehicle controls and treatments alone; the coloured bars indicate a significant additive increase in DNA damage in brown, a synergistic increase in DNA damage in yellow, a competitive antagonistic decrease in DNA damage in purple and an antagonistic decrease in DNA damage in pink. Statistical significance was set at *P*⩽0.05 compared with vehicle control, drugs alone and expected values of individual drugs effects.

Doxorubicin and etoposide when used in combination with quercetin or apigenin caused a synergistic increase in *γ*H2AX foci (*P*<0.05) in all lymphoid and myeloid cell lines (*P*<0.05) (Figure 6). However, when doxorubicin and etoposide were combined with emodin, rhein or *cis*-stilbene there was a synergistic increase in *γ*H2AX foci formation only in the lymphoid leukaemia cell lines (*P*<0.05) (Figure 6).

## Discussion

This study investigated the combination effects of two topoisomerase II inhibitors (doxorubicin and etoposide) and five polyphenols (quercetin, apigenin, emodin, rhein and *cis*-stilbene). These polyphenols have been shown to induce apoptosis and arrest cell cycle in leukaemia cell lines.^8^ Here, we demonstrated combination effects on ATP levels, induction of apoptosis and cell cycle arrest in leukaemia cell lines. Topoisomerase II inhibitor agents were synergistically enhanced by polyphenols in the lymphoid leukaemia cell lines, while their effects were differentially modulated by polyphenols in the myeloid leukaemia cell lines. These differential combination effects depended on cell lineage and/or polyphenol used. In the non-tumour control cells (CD34+HSC and CD133+HSC), all polyphenols were shown to reduce the toxicity of topoisomerase II inhibitors on ATP levels and caspase 3 activity, suggesting polyphenols could be protective in normal cells, and hence could reduce off-target effects.

Interestingly when doxorubicin and etoposide were used in combination with each polyphenol in lymphoid leukaemia cells they synergistically reduced ATP levels, induced apoptosis and increased cell cycle arrest, while in myeloid cell lines synergistic effects were only seen following combination treatments with quercetin and apigenin.

Quercetin has been shown in a number of solid tumours to enhance the activity of doxorubicin.^9–13^ Doxorubicin in combination with quercetin is highly effective against a number of tumour types including lymphoid and myeloid leukaemias. Doxorubicin and quercetin combination treatment in murine breast cancer cells caused synergistic inhibition of cell proliferation and metastasis to the lung, via increased IFN-*γ* and IL-2 levels and induction of immunogenic cancer cell death (apoptosis of CD4^+^ or CD8^+^ T cells).^11^ Similarly, in human breast cancer cell lines (MCF-7, MDA-MB-231 and MCF-10A) combination of doxorubicin and quercetin inhibited cell proliferation and induced apoptosis through decreased cellular thiol levels and blocked the PKC*δ* signalling pathway.^10^ The treatment of MCF-7 cells with doxorubicin and quercetin caused inhibition of cell proliferation and invasion via the suppression of hypoxia-inducible factor-1*α* and P-glycoprotein.^12^ Likewise, in human hepatoma cell lines (SMMC7721 and QGY7701) doxorubicin and quercetin combination treatment induced apoptosis via accumulation of p53, followed by the activation of mitochondrial apoptotic pathway, resulting in activation of caspase 9 and caspase 3.^13^ Furthermore, it has also been demonstrated that quercetin reduces the hepatotoxicity of doxorubicin in normal liver cells both *in vitro* and *in vivo*.^13^ Work by Brantley *et al.* ^13^ showed that doxorubicin synergistically interacted with apigenin in human breast cancer cell lines (MDA-MB-468 and MDA-MB-157) inhibiting cell proliferation and inducing apoptosis by altering the expression of apoptosis and proliferation markers such as BAX, Bcl-2, ERK, PARP and survivin.^14^ There is only one study which has previously investigated the effect polyphenols have on etoposide treatment; here quercetin acted synergistically with etoposide and inhibited cell proliferation and induced apoptosis in colorectal (HCT116) and prostate cancer (PPC1) cell lines, via upregulation of p53, p21 and BAX, and the reduction of cyclin B1 and survivin, which led to arrest of the cell cycle at S and G_2_/M phases.^15^ All these earlier studies are consistent with our findings, and suggest that the action of doxorubicin and etoposide are enhanced by both quercetin and apigenin.

In the current study, we further demonstrated synergistic responses were accompanied by a downregulation of glutathione levels and increased *γ*H2AX foci indicating DNA damage, driving apoptosis by a synergistic activation of caspase 9 and additive activation of caspase 8. These results suggest that synergistic effects are dependent on activation of caspase 9 in particular, suggesting a major role of intrinsic apoptosis and DNA damage response; however, as these cells are p53 null^16–18^ this could not be a direct regulation through the p53 pathway. Wang *et al*.,^12^ demonstrated that doxorubicin when combined with quercetin causes a synergistic induction of apoptosis through the accumulation of p53, and the activation of the intrinsic apoptotic pathway via the activation of caspase 9 and 3 in human hepatoma cell lines (SMMC7721 and QGY7701).^13^ Thus it is interesting that the intrinsic pathway is still activated even within the p53 null cell lines investigated in the current study.

Here we demonstrated that doxorubicin and etoposide combined with emodin, rhein or *cis*-stilbene in myeloid leukaemia cell lines had differential effects, with some being antagonistic. Doxorubicin when combined with emodin or rhein, and when etoposide was combined with rhein or *cis*-stilbene they induced antagonistic and/or competitive-antagonistic effects on ATP levels and apoptosis in the myeloid cell lines. These antagonistic combination treatments were usually associated with an elevation in glutathione levels and an absence of elevated DNA damage and cell cycle arrest.

Few studies have addressed polyphenol-induced antagonism of anti-tumour agents. Apigenin, galangin and chrysin have been shown to inhibit the action of cisplatin and doxorubicin, producing an antagonistic effect on cytotoxicity and induction of apoptosis in murine leukaemia (L1210) cells.^19^ It was suggested that this antagonistic effect was related to the antioxidant activity of the polyphenols, which could protect against reactive oxygen species (ROS) that were generated by doxorubicin. Similarly, rutin hydrate, quercetin dehydrate, hydrocaffeic acid, gallic acid and tannic acid antagonised bortezomib-induced apoptosis in multiple myeloma cell lines.^20^ Previous work has also shown that polyphenols can serve as either antioxidants or pro-oxidants depending on cellular conditions, requirement and doses; thus they can help in the modulation of antioxidant redox system such as glutathione.^21–23^ In addition, it has been reported that the antioxidant activity of polyphenols has a crucial role in their chemopreventive effect; while their pro-oxidant action may be important for their anti-cancerous effects.^21–23^ Here, for the first time we have shown the exact mechanisms of synergism and antagonism of topoisomerase inhibitors. The action of doxorubicin and etoposide, when combined with quercetin, apigenin, emodin, rhein or *cis*-stilbene was strongly dependent on the modulation of glutathione levels, caspase cascades and DNA damage within the leukaemia cell lines. These findings are supported by Staedler *et al*.^9^ who showed that doxorubicin when combined with quercetin in human breast cancer cell lines (MCF-7, MDA-MB-231 and MCF-10A) synergistically inhibited cell proliferation and induced apoptosis through the reduction of glutathione and glutathione S-transferase.^11^

Here, we have shown that the basal glutathione levels of the leukaemia cell lines strongly correlated with their sensitivity to both topoisomerase inhibitors and polyphenol treatments. It was found that the lymphoid leukaemia cell lines had the lowest basal glutathione levels and were more sensitive to polyphenols and topoisomerase inhibitor treatments when used alone or in combination. In contrast, the myeloid leukaemia cell lines had higher basal glutathione levels compared with non-tumour control cells and lymphoid cell lines. KG-1a myeloid cell line displayed the highest glutathione level and hence the greatest resistance to treatment with topoisomerase inhibitors and polyphenols.

Indeed, studies have reported that many cancers, including lung, ovarian, breast, colon, larynx and haematological malignancies have high levels of glutathione.^22^ This high glutathione level increases the antioxidant capacity of the cancerous cell preventing oxidative stress, DNA damage and inhibiting apoptosis; as a result this can lead to resistance to cancer treatments.^22^ This is also commonly associated with increased risk of disease relapse and resistance to the chemotherapeutics.^23,24^ In contrast, some cancers such as melanoma have low glutathione levels, which decrease the cellular antioxidant capacity and increased oxidative stress via excessive production of ROS, which can result in DNA damage and cell death; this increases the sensitivity of these cancers to treatment.^22^

The data reported in this study provides new and interesting information on the role of intracellular glutathione in the sensitization of leukaemia cells line to polyphenols and topoisomerase inhibitor treatment, whether used alone or in combination. Polyphenols/chemotherapy agents are more effective in cells with low glutathione content such as lymphoid cells, while less effective in cells with high glutathione content such myeloid cells. Cancer cell glutathione activity may predict responses to chemotherapy polyphenols combination treatments.

Currently, all the present studies reported that glutathione depletion in cells is strongly correlated to restored apoptosis induction, and this action could be very useful to increase the therapeutic efficacy of cancer treatment.^22,23,25^ It is reported that glutathione depletion is regulated by both extrinsic and intrinsic apoptotic signalling cascades at distinct checkpoints.^25^ In particularly, glutathione depletion can predispose cells to apoptosis or directly trigger cell death by modulation of both the permeability transition pore formation and the activation of execution caspases.^25^ It has also been reported that a reduction in the glutathione content is necessary for the formation of the apoptosome and activates the intrinsic apoptotic pathway by oxidation-dependent dimerisation.^25^ Glutathione depletion has also been shown to trigger cytochrome *c* release from the mitochondria, which can be oxidised for its pro-apoptotic action, which would need cytosolic glutathione levels to be depleted.^25^ The antioxidant property of glutathione is strongly linked to the overexpression of anti-apoptotic Bcl-2, which inhibits mitochondrial-induced apoptosis.^25^ Thus to increase the efficacy of chemotherapy or any cancer treatments, and limit multi-drug resistance (MDR), it is necessary to decrease glutathione levels in cancer cells.^22,26–28^

In those treatment combinations that induced synergistic accumulation in S and/or G_2_/M phases of the cell cycle, this was associated with *γ*H2AX foci formation and DNA damage. Alternatively in those combinations in which there were antagonistic effects there was no arrest of cell cycle and a lack of *γ*H2AX foci and DNA damage. Mechanistically, *γ*H2AX is phosphorylated by phosphatidylinositol-3 kinase (PI3K)-like kinases, including ATM, ATM-Rad3-related kinase (ATR), ATM-related kinase (ATX) and other cell cycle checkpoint factors such as Chk1 and Chk2.^29^ Thus the presence of *γ*H2AX foci is an excellent marker of DNA damage caused by cytotoxic agents or cancer treatments.^29–31^ Rajendran *et al.,* ^29^ suggested that combination treatments could be very effective and produced synergistic actions when used to target DNA damage and interfere with DNA repair in cancer cells. In this regard, our synergistic combination agents that targeted the DNA and cause damage and result in cell cycle arrest and/or apoptosis could be very effective treatments for leukaemia.

In conclusion, doxorubicin and etoposide activity can be enhanced by polyphenols, particularly in lymphoid leukaemia cells, although effects were strongly dependent on cell type, with some interactions being antagonistic in myeloid cell lines. These actions were strongly dependent on the modulation of glutathione levels and association with the DNA damage in leukaemia cell lines. Furthermore this study suggests that combination of doxorubicin and etoposide topoisomerase inhibitors with specific polyphenols could be promising for the treatment of lymphoid leukaemias. However, care is required to avoid potential inhibition of therapies.

## Materials and Methods

### Leukaemia cell lines

Four human leukaemia cell lines were used for this study: two lymphoid leukaemia cell lines (Jurkat (peripheral blood T cell leukaemia; ATCC: TIB-152, Middlesex, UK) and CCRF-CEM (acute lymphoblastic leukaemia; ATCC: CCL-119); which were previously shown to have high sensitivity to polyphenols;^8^ together with two myeloid leukaemia cell lines-THP-1 (acute monocytic leukaemia; ATCC: TIB-202) and KG-1a (acute myelogenous leukaemia; ATCC:CCL-243)-which had previously shown resistance to polyphenols.^8^ Non-tumour control cells from cord blood (CD34+HSC and CD133+HSC; Stem cell Technologies, Grenoble, France) were also included in the study. All cells were tested for mycoplasma contamination using the MycoAlert TM mycoplasma detection kit (Lonza, MD, USA) and were all negative throughout the study.

### Culture conditions

Cells were maintained in RPMI 1640 (Invitrogen, Paisley, UK) supplemented with 10% (v/v) foetal bovine serum, 1.5 mM L-Glutamine and 100 *μ*g/ml penicillin/streptomycin at 37 °C with 5% CO_2_.

### Treatment regimes

Quercetin (Enzo Life Sciences, Exeter, UK), apigenin, emodin, rhein and *cis*-stilbene (Sigma, Poole, UK) were prepared as described previously.^8^ Cells were treated with each polyphenol alone or in combination with the topoisomerase inhibitors (doxorubicin and etoposide; Sigma). Doxorubicin was dissolved in sterile distilled H_2_O, while etoposide was dissolved in 1 : 1 v/v sterile distilled H_2_O/ethanol. A stock solution of 25 mM was prepared with 10% (v/v) ethanol (Sigma) in serum-free media (Invitrogen) to generate treatment concentrations between 0.005 and 50 *μ*M.

Dose–response curves were generated for each polyphenol and each chemotherapy agent. These were used to determine the lowest-significant dose, which significantly reduced ATP levels and significantly induced caspase 3 activity when compared with the vehicle control at 24 h. These LSD doses were used for subsequent polyphenol/chemotherapy combination work. Significance was determined using a Kruskal–Wallis with a Conover–Inman *post hoc* test. The LSD for the selected polyphenols was determined from our previous study.^8^

The LSDs determined from effects on ATP levels were used in combination studies investigating effects on ATP levels, cell cycle progression, DNA damage and glutathione levels, while the LSDs determined from induction of apoptosis were used in combination studies investigating effects on induction of apoptosis, caspase 3, 8 and 9 activities.

Two lymphoid leukaemia cell lines (Jurkat and CCRF-CEM) and two myeloid leukaemia cell lines (THP-1 and KG-1a) and two non-tumour control cells (CD34+HSC and CD133+HSC) were treated with each polyphenol and each topoisomerase inhibitor (doxorubicin and etoposide) alone or in combination at their LSDs, along with a vehicle control, for 24 h. All treatments were performed in triplicate in three independent experiments. Following treatments, measurements were made of ATP levels, cell cycle progression, DNA damage (measured as *γ*H2AX foci), glutathione levels, induction of apoptosis and caspase 3, 8 and 9 activity.

### CellTiter-Glo luminescent cell viability assay

Cells were seeded into white 96-well plates (Fisher Scientific, Loughborough, UK) at 2.5×10^3^ cells per well and treated with polyphenols and topoisomerase inhibitors alone and in combination for 24 h, together with a 0.1% (v/v) ethanol vehicle controls. Following treatments, the CellTiter-Glo luminescent cell viability assay (Promega) was used to measure ATP levels, as per manufacturer's instructions.

### Apoptosis analysis

#### NucView caspase 3 activity assay by flow cytometry

Cells were seeded in 12-well plates at 0.5×10^6^ cells per well and treated with polyphenols and topoisomerase inhibitors alone or in combination for 24 h, together with a 0.1% (v/v) ethanol vehicle control. Following treatments, the NucView caspase 3 activity assay (Cambridge Bioscience, Cambridge, UK) was used to measure caspase 3 activity as per manufacturer's instructions. Samples were analysed by flow cytometry as described previously in the study by Mahbub *et al.* ^7^

#### Nuclear morphological analysis of apoptosis using double staining of Hoechst 33342/ propidium iodide and fluorescence microscopy

The effects of the combined topoisomerase inhibitor agents/polyphenols treatments were further investigated on the apoptotic nuclear morphological changes using Hoechst 33342 and propidium iodide staining and fluorescence microscopy (Sigma). Cells were seeded in 12-well plates at 0.5×10^6^ cells per ml and treated for 24 h with each topoisomerase inhibitor and each polyphenol alone and in combination at their LSD. A 0.1% (v/v) ethanol vehicle control was also included. Following 24 h of treatments, 100 *μ*l of cells were transferred to a 96-well plate, 10 *μ*l of 2 *μ*g/ml Hoechst 33342 dye was added to each well and incubated for 5 min in the dark. This was followed by the addition of 10 *μ*l of 2 *μ*g/ml propidium iodide dye and further incubated for 15 min in the dark. Plates were examined using inverted fluorescence microscope. Two hundred cells (live and apoptotic) were counted and the percentage of apoptotic nuclei determined for each sample. Images were captured from the fluorescent microscope using Cell-F software (Olympus).

### Cell cycle analysis using propidium iodide and flow cytometry

Cells were seeded in 12-well plates at 0.5×10^6^ cells per ml and treated with each topoisomerase inhibitor and polyphenols alone or in combination at their LSD for 24 h, together with a 0.1% (v/v) vehicle control. Following treatment, cells were harvested and cell cycle progression investigated using propidium iodide staining as described previously.^8^

### Caspases 8 and 9 Glo luminescent assays

Cells were seeded into white 96-well plates at 2.5×10^3^ cells per well and treated with each topoisomerase inhibitor and polyphenols alone and in combination at their LSD for 24 h, together with a 0.1% (v/v) vehicle control. Following treatment Caspase-Glo 8 and 9 assays were used as per manufacturer's instructions (Promega) to determine caspase 8 and 9 activity. Luminescence was measured using a Wallac Victor 2 1420 (Perkin Elmer Coventry, UK) and was normalised to vehicle controls.

### GSH-Glo glutathione assay

Basal glutathione levels were determined for the four leukaemia cell lines (CCRF-CEM, Jurkat, KG-1a and THP-1) and the two non-tumour control cells (CD133^+^ HSC and CD34^+^ HSC). Glutathione levels were also measured in all cells following treatment with polyphenols and topoisomerase inhibitors alone or in combination, for those treatments where combinations had previously shown a synergistic or antagonistic effect.

Cells were seeded into white 96-well plates at 2.5×10^3^ cells per well and treated with each topoisomerase inhibitor and polyphenols alone and in combination at their LSD for 24 h, together with a vehicle control. Following treatment, glutathione levels were measured using the GSH-Glo glutathione luminescent assay as per manufacturer's instructions. Luminescent signal was measured using a Wallac Victor 2 1420 and normalised to the vehicle control.

### DNA damage measured by *γ*H2AX foci detection

Alexa Fluor 647 Mouse anti-*γ*H2AX (pS139) (BD Pharmingen, Oxford, UK) is specifically designed for the phosphorylation of Ser-139 at the C-terminal region of *γ*H2AX enabling the visualisation of *γ*H2AX by immunofluorescence. The formation of *γ*H2AX foci indicates the presence of DNA damage. Cells were seeded into a BD Falcon 96-well Imaging Plate (BD Pharmingen) at 1.0×10^3^ cells per well and treated with each topoisomerase inhibitor and polyphenols alone and in combination at their LSD for 24 h, together with a vehicle control. Following treatments, cells were centrifuged at 400×*g* for 10 min then washed in PBS and fixed in BD Cytofix fixation buffer for 10 min (BD Pharmingen). The cells were then washed twice in PBS and permeabilised in 90% methanol (Sigma) for 5 min. Following washes, cells were incubated in 50 *μ*l Alexa Fluor 647 Mouse anti-*γ*H2AX (pS139) (1 : 10  v/v) at RT for 60 min in the dark. Following incubation, cells were washed three times in PBS and counter-stained in 100 *μ*l of 2 mg/ml Hoechst 33342 stain for 15 min. Cells were visualised using inverted fluorescence microscopy (Olympus, 1x2-UCB). Those cells with six and more *γ*-H2AX foci were considered as cells with DNA damage.^32^ The number of cells with substantial DNA damage (⩾6 foci) or without substantial DNA damage (<6 foci) were counted. At least 200 cells per sample were counted and percentage of cells with substantial DNA damage determined. Images were captured using an inverted fluorescence microscopy and the Cell-F software.

### Analysis of effects of combination treatments on ATP level, apoptosis, glutathione levels and DNA damage

The effects of the combination treatments were categorised using the following criteria:

- Reduction of ATP levels or induction of apoptosis or depletion of glutathione levels or induction of *γ*-H2AX foci formation by a polyphenol alone=X.
- Reduction of ATP levels or induction of apoptosis or depletion of glutathione levels or induction of *γ*-H2AX foci formation by chemotherapy agent alone=Y.
- Reduction of ATP levels or induction of apoptosis or depletion of glutathione levels or induction of *γ*-H2AX foci formation by combined polyphenol and chemotherapy agent treatment=Z.
- X+Y were calculated and described as our expected value.

The effect of combination chemotherapy and polyphenols treatments was classified as: additive or synergistic or competitive-antagonistic or antagonistic according to their statistical analysis using the Kruskal–Wallis and Conover–Inman *post hoc* tests. The combination effects were classified and defined as follows:

1. Additive: the effect of combination treatments (Z) is equal to the sum of the effect of the two treatments alone. This is an additive response if the combined effect (Z) is significantly greater than the effect of each treatment X and Y alone, as well as, significantly greater than the vehicle control, but not significantly greater than the expected value (X+Y).
2. Synergistic: the effect of combination treatments is higher than the sum of the effect of the two treatments alone. A synergistic response is seen if the combined effect (Z) is significantly greater than the vehicle control, each treatment alone (X alone, Y alone) and the expected value (X+Y).
3. Competitive-antagonistic: the effect of combination treatments is equal to the effect of one of two treatments. A competitive-antagonistic response is seen if the combined effect (Z) is significantly lower than the expected value (X+Y) and has a similar response to the effect of one treatment alone (X or Y alone) and is not significantly different.
4. Antagonistic: the effect of combination treatments is lower than the sum of the effect of the two treatments and individual treatments alone. An antagonistic response is seen if the combined effect (Z) is significantly lower than the effect of each treatment alone (X alone, Y alone) and the expected values (X+Y).

### Analysis of effect of combination treatments on cell cycle

The percentage of cells in each phase was analysed using the FlowJo software using the Watson pragmatic model. The data were expressed as medians with ranges (*n*=4). The statistical significance of individual drugs was determined first in comparison to the vehicle control using a Kruskal–Wallis and Conover–Inman *post hoc* tests. The statistical significance of combined drugs was determined in comparison to the vehicle control and individual treatments. The effect of combination treatments on cell cycle was classified either as: interactive, non-interactive or antagonistic. These classifications are defined as:

1. Interactive effect: the combination treatments induce a highly significant increase of cell accumulation in any phase of cell cycle, when compared with the vehicle control and those effects caused by the individual treatment alone.
2. Non-interactive effect: the combination treatments induced a significant increase of cell accumulation in any phase of cell cycle when compared with the vehicle control; but this was not significantly greater than that seen with one or both of treatments when used alone.
3. Antagonistic effect: the combination treatments had no significant difference in cell accumulation in any phase of cell cycle, when compared with the vehicle control, and was significant less when compared with the phases arrested by individual treatments alone.

### Statistical analysis

The median with range was calculated for the assays. Stats Direct software (Stats Direct, Altrincham, UK) was used to test whether data followed a normal distribution using a Shapiro–Wilke test, which was used to determine whether the data were parametric or non-parametric. As the data were non-parametric, a Kruskal–Wallis and Conover–Inman *post hoc* tests were used to determine statistical significance of the data. Results were considered statistically significant when *P*⩽0.05.

## Figures and Tables

**Table 1:** The effect of doxorubicin (DOX) and etoposide (ETP) on cell cycle progression, when used in combination with quercetin, apigenin, emodin, rhein, or cis-stilbene in two lymphoid leukaemia cell lines (Jurkat and CCRF-CEM) and two myeloid leukaemia cell lines (THP-1 and KG-1a)

	**Lymphoid leukaemia**				**Myeloid leukaemia**			
	**Jurkat**		**CCRF-CEM**		**THP-1**		**KG1a**	
	**DOX**	**ETP**	**DOX**	**ETP**	**DOX**	**ETP**	**DOX**	**ETP**
Quercetin	SYN interactive G_2_/M	SYN interactive G_2_/M	SYN interactive S	SYN interactive S & G2/M	SYN interactive S & G_2_/M	SYN interactive S & G_2_/M	Non-interactive S & G_2_/M	Non-interactive S & G_2_/M
Apigenin	SYN interactive G_2_/M	SYN interactive S	SYN interactive S & G_2_/M	SYN interactive S & G_2_/M	SYN interactive S & G_2_/M	SYN interactive S & G_2_/M	Non-interactive S & G_2_/M	SYN interactive S & G_2_/M
Emodin	SYN interactive G_2_/M	SYN interactive S & G_2_/M	SYN interactive S	SYN interactive S	ANTG no arrest	Non-interactive S & G_2_/M	ANTG no arrest	Non-interactive S & G_2_/M
Rhein	SYN interactive G_2_/M	SYN interactive G_2_/M	SYN interactive S	SYN interactive G_2_/M	ANTG no arrest	ANTG no arrest	ANTG no arrest	ANTG no arrest
*Cis*-Stilbene	SYN interactive G_2_/M	SYN interactive G_2_/M	SYN interactive S	SYN interactive S	SYN interactive S	ANTG no arrest	Non-interactive S & G_2_/M	ANTG no arrest

Cell cycle progression was analysed by flow cytometry following propidium iodide staining. Cells were treated with DOX or ETP and polyphenols alone and in combination for 24 h using their lowest-significant doses (LSD) as determined by CellTiter-Glo assay, together with a vehicle control. The percentage of cells in each phase was analysed with FlowJo software using the Watson (Pragmatic) model. The data were expressed as medians with ranges (*n*=4). Statistical significance of combination treatments was determined and compared with the vehicle control and the individual treatments alone. Statistical significance was set at *P*≤0.05. The combination effects of drugs were statistically shown to be either interactive or non-interactive, and shown to have a synergistic (SYN) or antagonistic effect (ANTG).

## Abbreviations

DOX: doxorubicin
ETP: etoposide
QUE: quercetin
AP: apigenin
EMO: emodin
RH: rhein
CIS: *cis*-stilbene.
LSD: lowest-significant dose.
